# Diagnosing a Gastric Submucosal Tumor Using Jumbo Biopsy “Unroofing” Technique: A Case Report and Review of the Literature

**DOI:** 10.1155/2013/414518

**Published:** 2013-11-14

**Authors:** Sakshi Kapur, Pradeep Mahal, Levin Miles, Adnan Hussain

**Affiliations:** ^1^Department of Internal Medicine, Overlook Medical Center, 99 Beauvoir Avenue, Summit, NJ 07902, USA; ^2^Division of Gastroenterology, Overlook Medical Center, Summit, NJ 07902, USA; ^3^Division of Pathology, Overlook Medical Center, Summit, NJ 07902, USA

## Abstract

We report a case of a 40-year-old female who presented with dyspeptic symptoms for six months. Upper GI endoscopy revealed a submucosal nodule in gastric antrum. Using “*jumbo biopsy unroofing technique*” we were able to get adequate tissue for analysis. Histopathology revealed a type III gastric carcinoid. Patient was treated with laparoscopic distal subtotal gastrectomy with Roux-en-Y reconstruction and partial omentectomy. Although there was no evidence of metastasis on initial presentation, eighteen months later, patient was found to have multiple metastatic lesions in her liver. Patient's lesions were treated with *intra-arterial (hepatic artery) Yttrium-90*.

## 1. Introduction

Gastric submucosal tumors are a common incidental finding occurring on routine upper GI endoscopies. Although different modalities for diagnosing these tumors are available, definitive diagnosis requires tissue analysis. Tissue acquisition for gastric submucosal tumors can be challenging. We report a case of a 40-year-old female who presented with dyspeptic symptoms. Upper GI endoscopy revealed a submucosal nodule in gastric antrum. Histopathology was compatible with a type III gastric carcinoid. Although there was no evidence of metastasis on initial presentation, eighteen months later, patient was found to have multiple metastatic lesions in her liver. Our case highlights the malignant potential of a gastric submucosal nodule, which may otherwise present as an incidental finding on upper GI endoscopy.

## 2. Case Presentation

A 40-year-old female presented with dyspeptic symptoms for six months. She denied any nausea, vomiting, or change in bowel habits. There was no history of hematemesis, melena, or weight loss. Physical examination revealed an obese female with normal vital signs. Head and neck exam was positive for mild pallor but no icterus, thyromegaly, or lymph node enlargement was noted. Abdomen was soft, nontender with no hepatosplenomegaly.

Workup revealed a Hb of 11 gm/dL, white blood count of 11000/*μ*L, and a platelet count of 2.26 × 10^3^/*μ*L. Blood urea nitrogen, creatinine, and electrolytes were normal.

**Figure 1:**
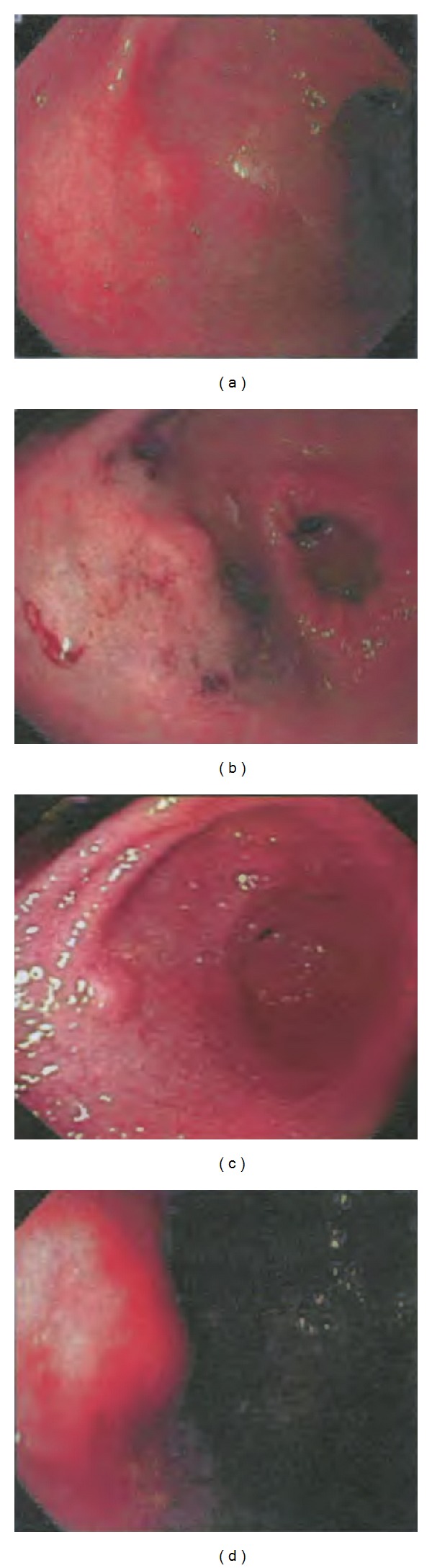
Esophagogastroduodenoscopy showing a submucosal nodule in gastric antrum.

Patient underwent an esophagogastroduodenoscopy and was found to have a *Helicobacter pylori* related chronic active gastritis. Concurrently, a submucosal mass measuring about 2.0 cm was noted in the gastric antrum (Figure 1).

**Figure 2:**
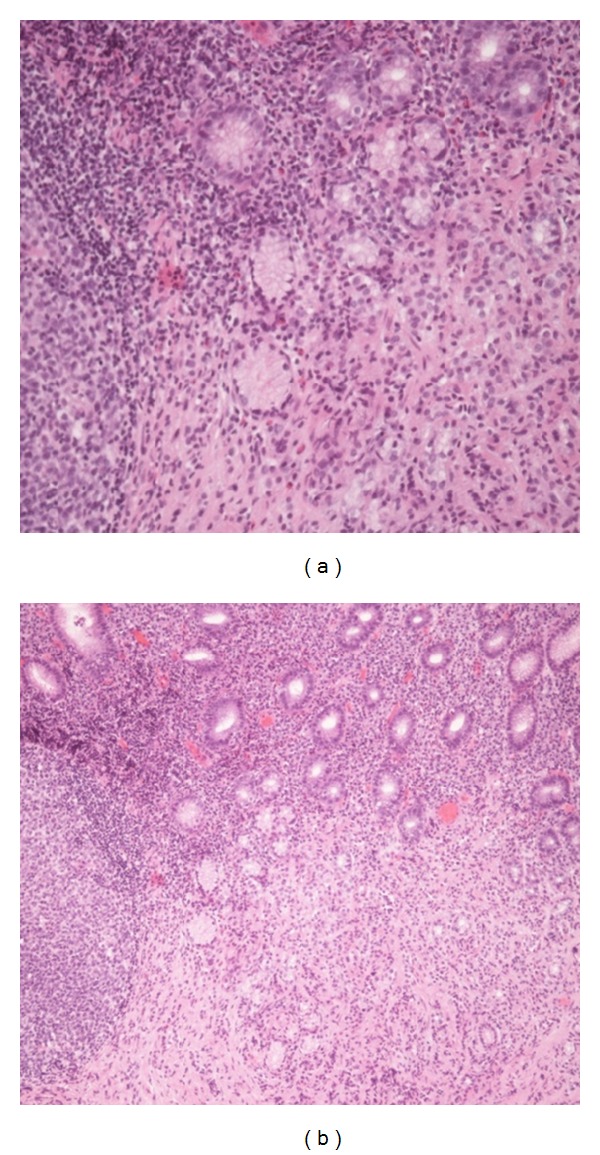
A well-differentiated neuroendocrine tumor consistent with carcinoid seen.

**Figure 3:**
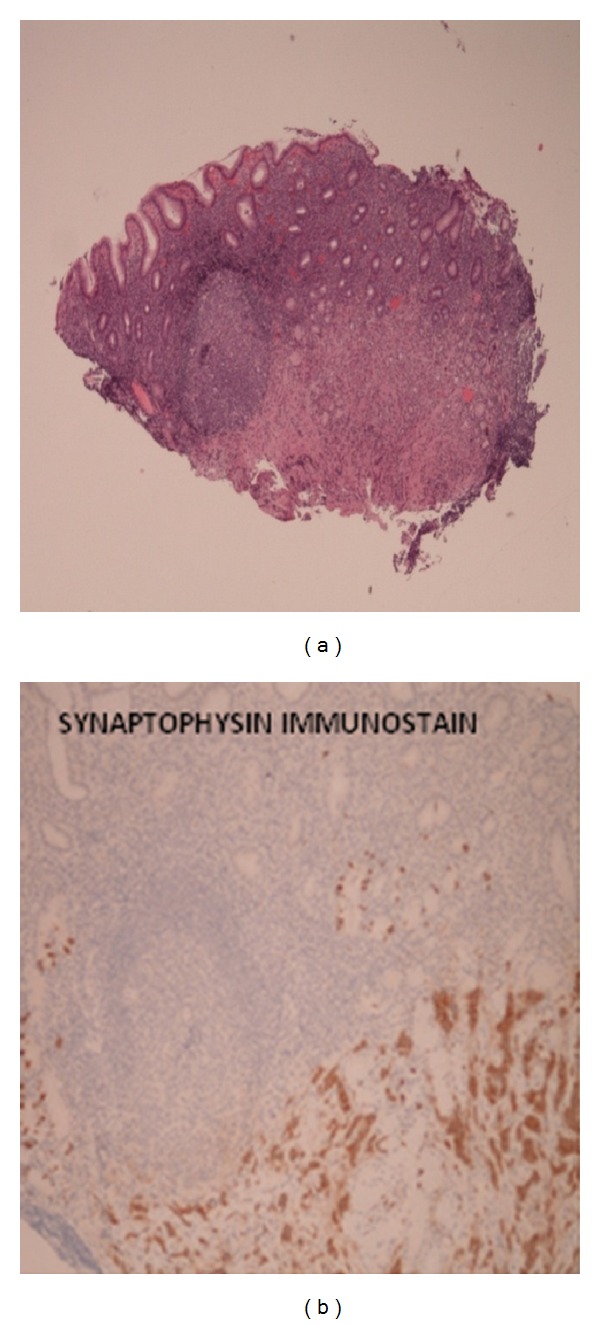
A well-differentiated neuroendocrine tumor consistent with carcinoid seen.

Using jumbo biopsy “unroofing” technique, we were able to get extensive biopsies of this mass, and results revealed a well differentiated neuroendocrine tumor (NET) consistent with a gastric carcinoid (Figures 2 and 3). Subtyping confirmed a type III gastric carcinoid.

Subsequently, an endoscopic ultrasound (EUS) was done that confirmed a submucosal mass slightly smaller in size than initially anticipated, arising from the third layer, and no lymphadenopathy. Biomarkers for carcinoid such as serotonin, chromogranin A, and 5-HIAA were also negative. Computer tomography of the abdomen revealed gastric antral thickening secondary to carcinoid and no evidence of extragastric extension, liver, or adrenal metastasis.

**Figure 4:**
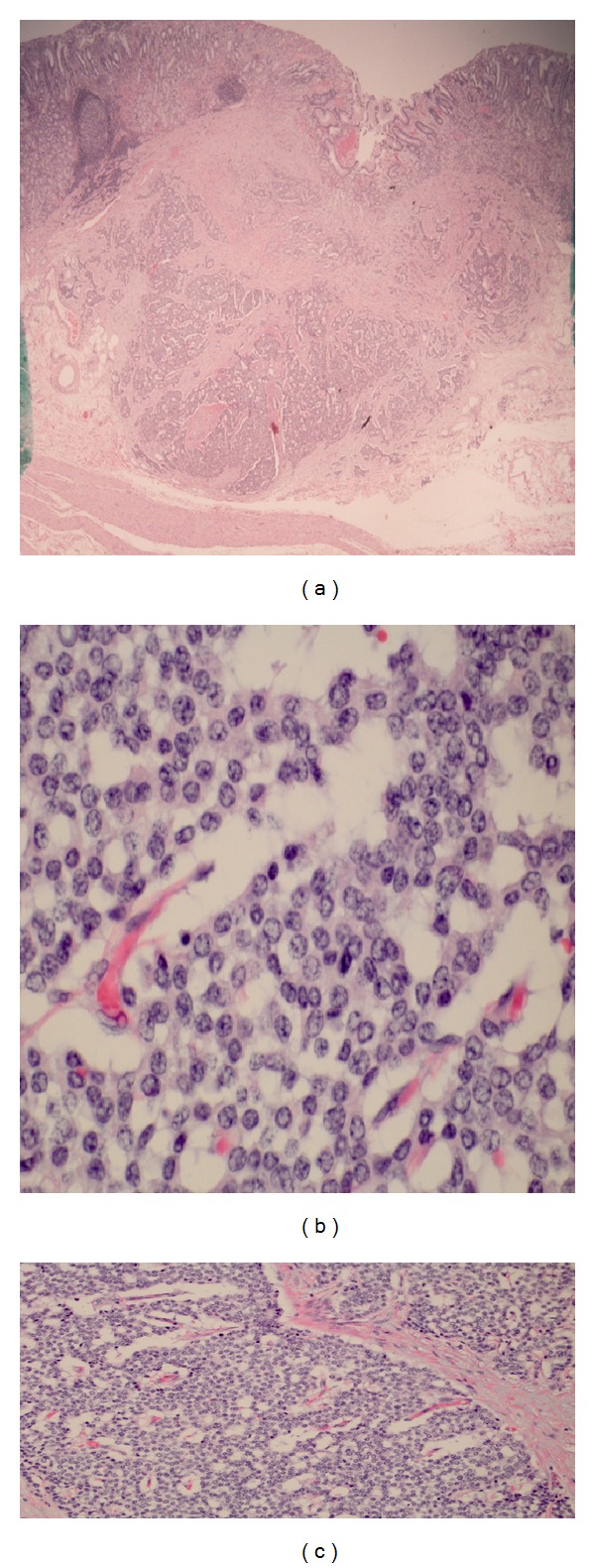
A well-differentiated neuroendocrine tumor consistent with a gastric carcinoid.

Patient was treated with laparoscopic distal subtotal gastrectomy with Roux-en-Y reconstruction and partial omentectomy. Biopsy results confirmed a well-differentiated neuroendocrine tumor (NET) consistent with type III gastric carcinoid, restricted to submucosa, without involvement of the muscularis propria (Figure 4). Twenty-two lymph nodes (17 in greater curvature and 5 in lesser curvature) were negative for metastasis, and the omentum was also benign.

**Figure 5:**
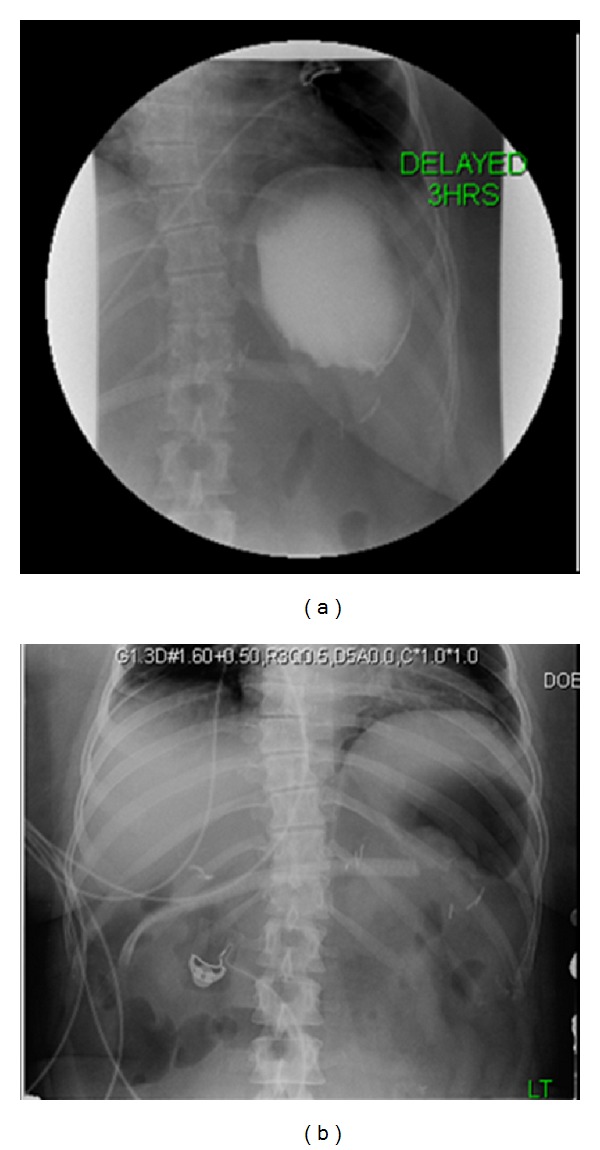
Contrast seen flowing from distal esophagus into remaining stomach without leakage from the anastomotic site.

Postoperatively, patient did well but complained of some nausea. An upper gastrointestinal series was performed, which ruled out leakage from the anastomotic site (Figure 5). Patient tolerated the diet well and was discharged from the hospital.

**Figure 6:**
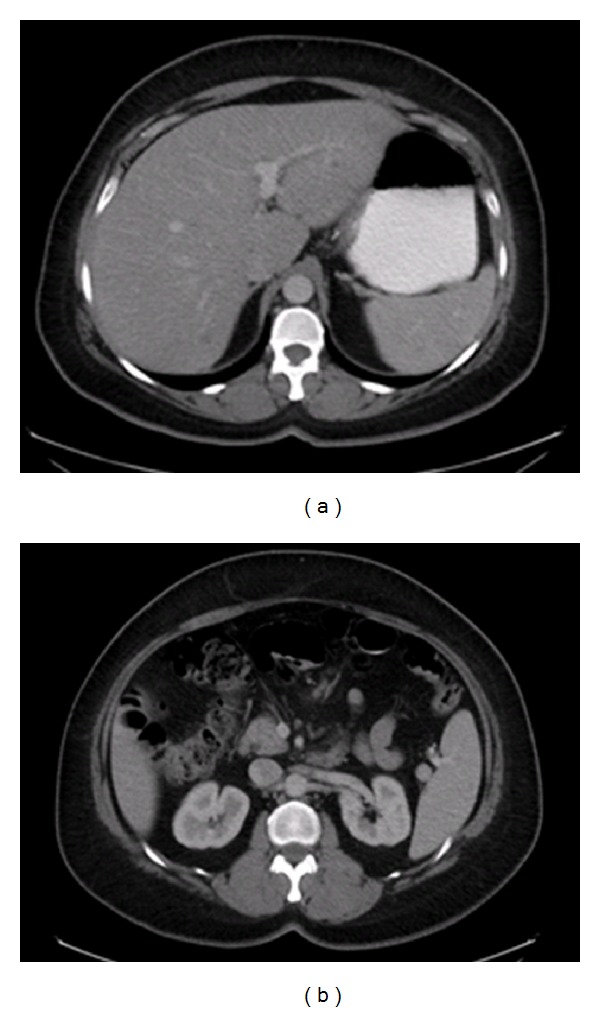
Heterogeneous liver with multiple low attenuating foci and a soft tissue density seen in small bowel mesentery.

Eighteen months later, patient presented to the hospital with progressively worsening generalized abdominal pain for one month. She complained of occasional nausea but denied any change in her appetite or weight. Physical examination was unremarkable. Computer tomography of the abdomen showed multiple, small, ill-defined, and low attenuating lesions in the left lobe of liver and a 1.7 cm mass in small bowel mesentery (Figure 6).

**Figure 7:**
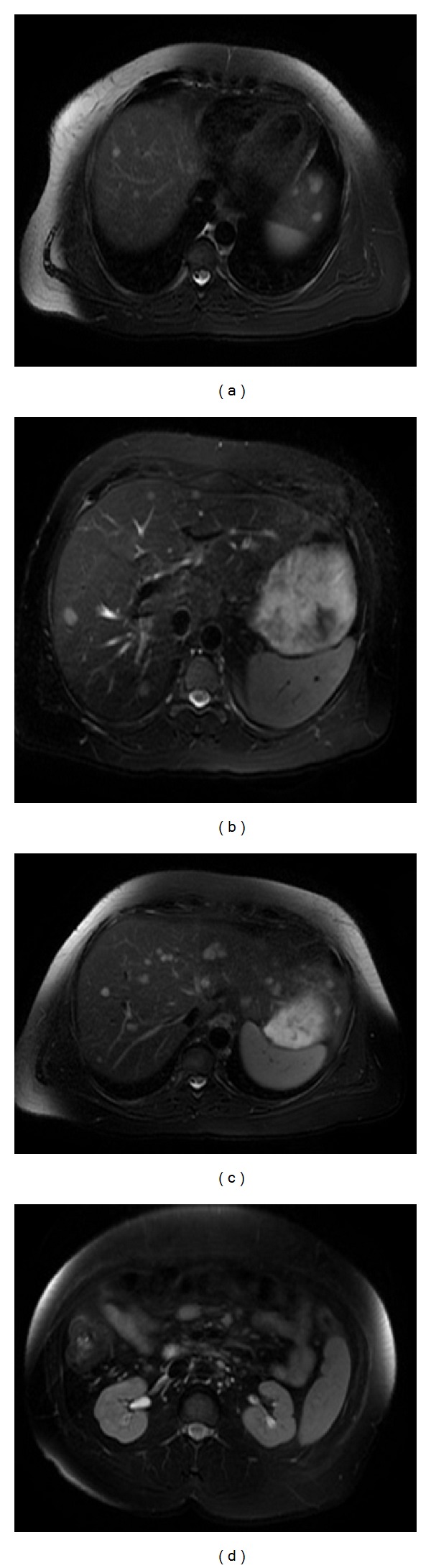
Numerous hepatic lesions seen, with the largest lesion at the dome of the lateral segment of left lobe of liver measuring 2.1 × 1.4 cm; multiple enlarged lymph nodes in small bowel mesentery and porta hepatis also seen.

Magnetic resonance imaging revealed several hepatic lesions, with the largest lesion measuring 2.1 × 1.4 cm in left lobe of liver. Multiple lymph nodes in small bowel mesentery and porta hepatis were also enlarged (Figure 7).

**Figure 8:**
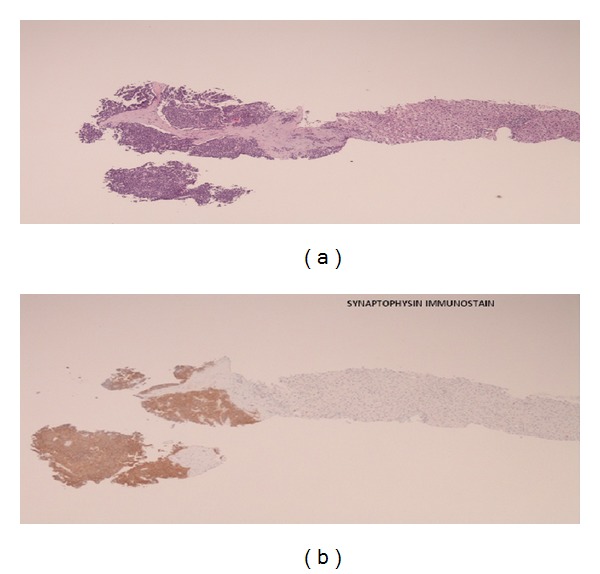
Liver biopsy consistent with metastatic gastric carcinoid.

A computer tomography guided core biopsy of hepatic lesions was performed, and results confirmed metastatic lesions, secondary to gastric carcinoid (Figure 8). Patient was treated with intra-arterial (hepatic artery) Yttrium-90.

Patient has been following with us for over two years, and her lesions have been stable so far.

## 3. Discussion

Gastric submucosal tumors (SMTs) are a common incidental finding occurring on routine upper GI endoscopies. The exact prevalence of these lesions is uncertain, although one retrospective study reported an incidence of 0.36% [1].

The differential ranges from benign lesions such as fibroma, lipoma, leiomyoma, varices, and heterotopic pancreas to malignant or potentially malignant lesions like lymphoma, gastrointestinal stromal tumors (GISTs), carcinoid, neurofibroma, schwannoma, and so forth. Extraluminal compression secondary to visceral structures can also appear as a submucosal nodule on endoscopy. The most common source of extraluminal compression is from spleen and splenic vessels [2]. Although the differential is very wide, definitive diagnosis depends upon tissue histopathology.

Certain risk criteria for malignancy in a submucosal nodule have been established on EUS. These include size > 3 cm, inhomogeneous echo pattern, irregular margins, and presence of lymph nodes [3]. EUS has 64% sensitivity and 80% specificity in diagnosing malignant SMTs, when at least two of these criteria are present [4]. Lesions such as lipoma, heterotopic pancreas, and duplication cyst have a characteristic appearance on EUS [3]. However, hypoechoic lesions such as leiomyoma, GIST, carcinoid, and schwannomas need tissue diagnosis for confirmation. EUS can differentiate a real SMT from extraluminal compression caused by visceral organs. EUS examination of the gastric wall typically exhibits five layers. EUS can also help us determine the exact layer of origin of a SMT. Lesions arising from third and fourth echo layers have a high probability of being malignant and warrant a tissue diagnosis.

Cross-sectional imaging like CT and MRI can be used to evaluate the extent of metastasis for malignant tumors like GIST [5]. However, cross-sectional imaging unlike EUS does not offer the advantage of identifying the exact layer of origin of a submucosal nodule.

Gastric carcinoids are rare lesions that constitute less than 1% of all stomach neoplasms. They arise from enterochromaffin-like cells lining the gastric mucosa. Carcinoids can be subtyped into three categories. Type I and type II carcinoid account for 80% of these lesions and are associated with atrophic gastritis and Zollinger Ellison syndrome respectively. Management includes endoscopic resection followed by endoscopic surveillance. Type III carcinoid accounts for 15–20% of these tumors and has the highest rate of metastases (>50%). Most of these tumors are metastatic at the time of presentation. Treatment of this subtype requires surgical resection. On endoscopy, carcinoids appear as polyploidal masses with a normal overlying mucosa [6]. They usually arise from second or third layer on EUS. Adequate sampling and subsequent subtyping not only help us in making a definitive diagnosis, but also help us to choose the correct treatment modality for these patients.

Gastrointestinal stromal tumors (GISTs) are the most common mesenchymal tumors of the GI tract. The annual incidence is estimated to be at least 10 to 20 cases per million [7]. Most GISTs are positive for C-KIT and CD 34 staining and are thought to arise from interstitial cell of Cajal. GI autonomic nerve tumors (GANTs) are also categorized under GISTS owing to their immunohistochemical resemblance. 65% of the GISTS occur in stomach and appear as submucosal nodules on upper GI endoscopy. Up to 10% to 30% of GISTS are malignant. However, recent data suggests that all GISTs have a malignant potential [8]. On EUS examination, GISTs typically arise from the fourth layer, and size, irregular borders, lobulation, and echogenic foci indicate malignancy [9]. Endoscopic differentials include gastric lymphoma and inflammatory fibroid polyp [10]. Therefore, tissue diagnosis of a submucosal nodule should not only be able to differentiate a GIST from a non-GIST, but also evaluate the malignant potential of this tumor.

Tissue acquisition for a gastric SMT can be challenging. The yield of standard endoscopy is usually poor [1]. EUS guided fine needle aspiration (FNA) can be used to obtain tissue, but the yield is often inadequate to make a definitive diagnosis, especially for mesenchymal tumors and when differentiation between benign and malignant stromal tumors is needed [11]. Tissue yield of EUS-FNA ranges from 50% to 93% [11, 12]. In a prospective study by Turhan et al., the sensitivity, specificity, positive and negative predictive values, and accuracy of EUS-FNA for diagnosing submucosal mesenchymal tumors of upper GI tract were 82.9%, 73.3%, 87.9%, 64.7%, and 80%, respectively. The corresponding values for nonmesenchymal lesions were 100%, 85.7%, 80%, 100%, and 90.9% [13].

Although cytological examination is usually sufficient to make a diagnosis of GIST, differentiation between benign and malignant stromal tumors requires histopathological and immunochemical analysis. Pathological assessment of GIST requires immunohistochemical staining for c-KIT (CD 117). 95% of GISTs are positive for C-KIT. Immunohistochemical stains can also be used to differentiate GISTs from endoscopic differentials like lymphoma and inflammatory polyp. However, there are some GISTs that are negative for C-KIT. In these tumors, DOG 1 gene expression can be used. DOG 1 has a greater sensitivity as compared to C-KIT [14, 15]. Ki-67 labeling index has also been used to differentiate benign from malignant GISTs. In a study conducted by Ando et al. and Liu et al., the accuracy for Ki-67 in predicting the aggressiveness of GIST was over 90% [16, 17].

Various factors such as size of lesion, site (lesions in lower third of stomach are difficult to sample), number of needle passes, on-site cytopathologist, and cytological versus histopathological assessment can affect the outcome of EUS-FNA. Side effects of EUS-FNA include bleeding and infections. EUS Doppler before EUS-FNA can prevent rupture of a varix, which otherwise might be mistaken for a submucosal nodule [18].

EUS guided trucut needle biopsy (TCB) has been used for acquisition of core tissue specimens. Procedural difficulties such as needle stiffness and lesions in distal stomach can pose challenge for an endoscopist. The combination of both EUS-FNA and EUS TCB has been found to be superior to either technique alone [19]. Combining these two methods has shown to increase the diagnostic accuracy to 95%, without an immediate cytopathologist [19].

Jumbo biopsy forceps can be used for obtaining tissue from deeper layers of the gastric wall. In a retrospective study by Buscaglia et al., out of the 129 patients with subepithelial lesions of the upper and lower GI tract that underwent EUS with biopsy using jumbo forceps, 58.9% of patients had a definitive diagnosis [20]. The results in third layer (EUS) were the most definitive. However, 34.9% of patients experienced significant bleeding and required some form of endoscopic hemostasis [20]. In another study by Komanduri et al., out of the 66 patients that underwent jumbo biopsy “unroofing technique” for tissue acquisition, 92% provided adequate tissue, without significant complications [21, 22]. Jumbo biopsy forceps along with on-site “touch preparation cytology” have shown to further increase the accuracy [22]. Therefore, use of jumbo biopsy forceps for tissue acquisition seems to be a safe and effective option for diagnosing gastric SMTs.

## 4. Conclusion

Tissue diagnosis of a gastric SMT can be challenging. Use of jumbo biopsy “unroofing technique” seems to be an attractive option for diagnosing these tumors. On-site “touch preparation cytology” has shown to further increase the accuracy.
